# Supplementary material for: Experimental evolution to thermal stress indicates climate resilience in a cosmopolitan arthropod
Source: eLife. 2026 Jun 30;15:RP110352. doi: 10.7554/eLife.110352 (PMC13318303; doi:10.7554/eLife.110352)
Supplement: MDAR checklist [file elife-110352-mdarchecklist1.docx]

**Materials Design Analysis Reporting (MDAR)**

**Checklist for Authors**

The MDAR framework establishes a minimum set of requirements in transparent reporting mainly applicable to studies in the life sciences.

*eLife* asks authors to **provide detailed information within their article** to facilitate the interpretation and replication of their work. Authors can also upload supporting materials to comply with relevant reporting guidelines for health- related research (see EQUATOR Network), life science research (see the BioSharing Information Resource), or animal research (see the ARRIVE Guidelines and the STRANGE Framework; for details, see *eLife*’s Journal Policies). Where

applicable, authors should refer to any relevant reporting standards materials in this form.

For all that apply, please note **where in the article** the information is provided. Please note that we also collect information about data availability and ethics in the submission form.

**Materials:**

| **Newly created materials** | **Indicate where provided: section/figure legend** | **N/A** |
| --- | --- | --- |
|  |  |  |
| The manuscript includes a dedicated "materials availability statement" providing transparent disclosure about availability of newly created  materials including details on how materials can be accessed and describing any restrictions on access. | Materials and Methods → "Insects" section; Key Resources Table. Newly generated strains (HS, CS, SODC-MU1, SODC-MU2, SODC-MU3) are available from the corresponding author upon reasonable request. |  |

| **Antibodies** | **Indicate where provided: section/figure legend** | **N/A** |
| --- | --- | --- |
|  |  |  |
| For commercial reagents, provide supplier name, catalogue number and RRID, if available. |  | N/A |

| **DNA and RNA sequences** | **Indicate where provided: section/figure legend** | **N/A** |
| --- | --- | --- |
|  |  |  |
| Short novel DNA or RNA including primers, probes: Sequences should be included or deposited in a public repository. | Supplementary File 7 (full list of primers, sgRNAs, and dsRNA sequences); Materials and Methods → "Gene cloning", "Deletion of the targeted gene using the CRISPR/Cas9 genome editing", and "RNA interference mediated silencing of target genes". |  |

| **Cell materials** | | **Indicate where provided: section/figure legend** | | **N/A** | |
| --- | --- | --- | --- | --- | --- |
|  | |  | |  | |
| Cell lines: Provide species information, strain. Provide accession number in repository OR supplier name, catalog number, clone number, OR  RRID. | |  | | N/A | |
| Primary cultures: Provide species, strain, sex of origin, genetic modification status. | |  | | N/A | |
| **Experimental animals** | | **Indicate where provided: section/figure legend** | | **N/A** | |
|  | |  | |  | |
| Laboratory animals or Model organisms: Provide species, strain, sex,  age, genetic modification status. Provide accession number in  repository OR supplier name, catalog number, clone number, OR RRID. | | Materials and Methods → "Insects" section; Key Resources Table. *Plutella xylostella* (Lepidoptera: Plutellidae); ancestral, hot, cold, and three SODC-MU strains; both sexes; all developmental stages used as indicated. | |  | |
| Animal observed in or captured from the field: Provide species, sex, and age where possible. | | Materials and Methods → "Insects" section. Founding population of *P. xylostella* collected from organic cabbage farms in Fuzhou, Fujian Province, China (26°05'N, 119°18'E) in July 2012; both sexes and multiple larval instars included. | |  | |

| **Plants and microbes** | **Indicate where provided: section/figure legend** | **N/A** |
| --- | --- | --- |
|  |  |  |
| Plants: provide species and strain, ecotype and cultivar where relevant, unique accession number if available, and source (including location for collected wild specimens). | Materials and Methods → "Insects" section. Cabbage (*Brassica oleracea* var. capitata) used as the host plant for founding population collection in Fuzhou, China. |  |
| Microbes: provide species and strain, unique accession number if available, and source. | Materials and Methods *E. coli* DH5α competent cells (YEASEN, Shanghai, China) used as cloning host. |  |

| **Human research participants** | **Indicate where provided:**  **section/figure legend) or state if these demographics were not**  **collected** | **N/A** |
| --- | --- | --- |
|  |  |  |
| If collected and within the bounds of privacy constraints report on age, sex, gender and ethnicity for all study participants. |  | N/A |

**Design:**

| **Study protocol** | **Indicate where provided: section/figure legend** | **N/A** |
| --- | --- | --- |
|  |  |  |
| If the study protocol has been pre-registered, provide DOI. For clinical trials, provide the trial registration number OR cite DOI. |  | N/A |

| **Laboratory protocol** | | **Indicate where provided: section/figure legend** | | **N/A** | |
| --- | --- | --- | --- | --- | --- |
|  | |  | |  | |
| Provide DOI OR other citation details if detailed step-by-step protocols are available. | | All protocols are described in detail in Materials and Methods. No separate protocols.io DOI deposited. | |  | |
| **Experimental study design (statistics details)** | | | | | |
| **For in vivo studies: State whether and how the following have been done** | | **Indicate where provided:**  **section/figure legend. If it could have been done, but was not, write “not done”** | | **N/A** | |
| Sample size determination | | Materials and Methods → "Data analysis" and individual figure legends. Sample sizes were chosen based on standard practice in insect experimental evolution studies and our prior work (Lei et al., 2024). | |  | |
| Randomisation | | Materials and Methods → "Insects" and "Development of age-stage-specific two-sex life tables". Eggs, nymphs, pupae, and adults were randomly selected for all assays; replicate populations were randomly assigned to thermal regimes at the start of the selection experiment. | |  | |
| Blinding | | Not done. Blinding was not performed because experimenters needed to identify strains and treatments. Quantitative measurements (qPCR, enzyme activity, supercooling/freezing points, CTmax) were instrument-based, minimizing experimenter bias. | |  | |
| Inclusion/exclusion criteria | | Materials and Methods. Pre-established criteria: only synchronized newly laid eggs / newly emerged adults were included; only homozygous CRISPR/Cas9 mutants confirmed by Sanger sequencing were retained. No data points were excluded post-hoc. | |  | |

| **Sample definition and in.laboratory replication** | **Indicate where provided: section/figure legend** | **N/A** |
| --- | --- | --- |
|  |  |  |
| State number of times the experiment was replicated in the laboratory. | Individual figure legends and Materials and Methods. Number of biological replicates is specified for every experiment (e.g., n = 3 for transcriptomics; n = 4 for SOD/O2- assays; n = 6 for thermal tolerance; n = 40 for supercooling/freezing points). |  |
| Define whether data describe technical or biological replicates. | Defined in figure legends and Materials and Methods → "qRT-PCR analysis". Biological replicate = independent cohort/pool of insects; technical replicate = repeated measurement of same biological sample (3 technical replicates per biological sample in qRT-PCR). |  |

| **Ethics** | **Indicate where provided: section/submission form** | **N/A** |
| --- | --- | --- |
|  |  |  |
| Studies involving human participants: State details of authority granting ethics approval (IRB or equivalent committee(s), provide reference  number for approval. |  | N/A |
| Studies involving experimental animals: State details of authority granting ethics approval (IRB or equivalent committee(s), provide reference number for approval. |  | N/A |
| Studies involving specimen and field samples: State if relevant permits obtained, provide details of authority approving study; if none were  required, explain why. | Materials and Methods → "Insects" section. Field collection conducted with permission of cabbage farm owners in Fuzhou, China. *P. xylostella* is a widespread agricultural pest not listed under any conservation regulation; no special permits required. |  |

| **Dual Use Research of Concern (DURC)** | **Indicate where provided: section/submission form** | **N/A** |
| --- | --- | --- |
|  |  |  |
| If study is subject to dual use research of concern regulations, state the authority granting approval and reference number for the regulatory  approval. |  | N/A |

**Analysis:**

| **Attrition** | **Indicate where provided: section/figure legend** | **N/A** |
| --- | --- | --- |
| Describe whether exclusion criteria were pre-established. Report if  sample or data points were omitted from analysis. If yes, report if this was due to attrition or intentional exclusion and provide justification. | Materials and Methods and figure legends. Exclusion criteria pre-established. CRISPR/Cas9: of 162 injected eggs, 75 reached adulthood; only homozygous mutants (verified by Sanger sequencing) were retained. No outliers removed from quantitative datasets. |  |

| **Statistics** | **Indicate where provided: section/figure legend** | **N/A** |
| --- | --- | --- |
|  |  |  |
| Describe statistical tests used and justify choice of tests. | Materials and Methods → "Data analysis" section and individual figure legends. Shapiro-Wilk normality test; t-test or Mann-Whitney for two groups; one-way ANOVA + Tukey's HSD or Tamhane's T2, or Kruskal-Wallis for multiple groups; paired bootstrap (100,000 replicates) for life table parameters; Benjamini-Hochberg FDR for omics. SPSS v23.0. *P* < 0.05 considered significant. |  |

| **Data availability** | **Indicate where provided: section/submission form** | **N/A** |
| --- | --- | --- |
|  |  |  |
| For newly created and reused datasets, the manuscript includes a data availability statement that provides details for access (or notes  restrictions on access). | Data availability statement (after Additional files). Raw sequencing data: GSA accession CRA024611. Metabolomic data: OMIX009807 and OMIX009846. All other data are included in the manuscript, supplementary files, and source data files. |  |
| When newly created datasets are publicly available, provide accession number in repository OR DOI and licensing details where available. | Data availability statement. GSA: CRA024611 (https://ngdc.cncb.ac.cn/gsa); OMIX: OMIX009807 (https://ngdc.cncb.ac.cn/omix/preview/kkl093nl) and OMIX009846 (https://ngdc.cncb.ac.cn/omix/preview/gXwZCXMI). |  |
| If reused data is publicly available provide accession number in repository OR DOI, OR URL, OR citation. |  | N/A |

| **Code availability** | | **Indicate where provided: section/figure legend** | | **N/A** |
| --- | --- | --- | --- | --- |
| For any computer code/software/mathematical algorithms essential for replicating the main findings of the study, whether newly generated or re-used, the manuscript includes a data availability statement that  provides details for access or notes restrictions. | |  | | N/A |
| Where newly generated code is publicly available, provide accession number in repository, OR DOI OR URL and licensing details where  available. State any restrictions on code availability or accessibility. | |  | | N/A |
| If reused code is publicly available provide accession number in repository OR DOI OR URL, OR citation. | Key Resources Table and Materials and Methods. Software used: HISAT2 v2.1.0 (RRID:SCR_015530); DESeq2 (RRID:SCR_015687); WGCNA v2.0 (RRID:SCR_003302); GROMACS 2022.3 (RRID:SCR_014565); AlphaFold2 (RRID:SCR_025089); IQ-TREE (RRID:SCR_017254); SPSS v23.0 (RRID:SCR_002865); TWOSEX-MSChart (Chi et al., 2020). | |  | |

**Reporting:**

The MDAR framework recommends adoption of discipline-specific guidelines, established and endorsed through community initiatives.

| **Adherence to community standards** | **Indicate where provided: section/figure legend** | **N/A** |
| --- | --- | --- |
|  |  |  |
| State if relevant guidelines (e.g., ICMJE, MIBBI, ARRIVE, STRANGE) have been followed, and whether a checklist (e.g., CONSORT, PRISMA,  ARRIVE) is provided with the manuscript. |  | N/A |


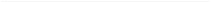


* We provide the following guidance regarding transparent reporting and statistics; we also refer authors to Ten common statistical mistakes to watch out for when writing or reviewing a manuscript.

**Sample-size estimation**

● You should state whether an appropriate sample size was computed when the study was being designed

● You should state the statistical method of sample size computation and any required assumptions

● If no explicit power analysis was used, you should describe how you decided what sample (replicate) size (number) to use

**Replicates**

● You should report how often each experiment was performed

● You should include a definition of biological versus technical replication

● The data obtained should be provided and sufficient information should be provided to indicate the number of independent biological and/or technical replicates

● If you encountered any outliers, you should describe how these were handled

● Criteria for exclusion/inclusion of data should be clearly stated

● High-throughput sequence data should be uploaded before submission, with a private link for reviewers provided (these are available from both GEO and ArrayExpress)

**Statistical reporting**

● Statistical analysis methods should be described and justified

● Raw data should be presented in figures whenever informative to do so (typically when N per group is less than 10)

● For each experiment, you should identify the statistical tests used, exact values of N, definitions of center, methods of multiple test correction, and dispersion and precision measures (e.g., mean,

median, SD, SEM, confidence intervals; and, for the major substantive results, a measure of effect size (e.g., Pearson's r, Cohen's d)

● Report exact p-values wherever possible alongside the summary statistics and 95% confidence

intervals. These should be reported for all key questions and not only when the p-value is less than 0.05.

**Group allocation**

● Indicate how samples were allocated into experimental groups (in the case of clinical studies, please specify allocation to treatment method); if randomization was used, please also state if restricted randomization was applied

● Indicate if masking was used during group allocation, data collection and/or data analysis
