# Supplementary material for: Experimental evolution to thermal stress indicates climate resilience in a cosmopolitan arthropod
Source: eLife. 2026 Jun 30;15:RP110352. doi: 10.7554/eLife.110352 (PMC13318303; doi:10.7554/eLife.110352)
Supplement: Peer review history

# Editor report: eLife Assessment

**DOI:** 10.7554/eLife.110352.3.sa0

**Authors:** Ariel D Chipman

**Affiliations:** The Hebrew University of Jerusalem Israel

**Keywords:** Convincing, Important

This **important** study deepens our understanding of how populations of a given species may diverge in their molecular and physiological patterns as a result of adaptation to different thermal regimes. By approaching this question from multiple directions, the authors provide **convincing** evidence for adaptive changes in three strains of the diamondback moth after only three years of experimental evolution. This work will be of interest to anyone working on the response of pest species to environmental change and to workers on adaptive evolution in general.

# Referee report: Reviewer #1 (Public review)

**DOI:** 10.7554/eLife.110352.3.sa1

Summary:

In this manuscript, Lei and co-workers aim to uncover the genetic underpinnings of thermal adaptation across three strains of the diamondback moth (Plutella xylostella) through experimental evolution over three years under three different thermal regimes. They identify systematic differences in trait responses (e.g., survival, fecundity), metabolic profiles, gene expression, and in the amino acid sequence of the PxSODC gene, among others. These results suggest that the diamondback moth has a strong potential for rapid physiological adaptation to different thermal regimes. Overall, this is a comprehensive and generally well-executed study that addresses an important question in the face of ongoing climate change.

Strengths:

The authors employ multiple approaches to identify signatures of thermal adaptation across the three strains, such as trait performance comparisons, metabolomics, transcriptomics, and amino acid sequence comparisons. All these different angles form a convincing picture of the underlying factors that underpin thermal adaptation in this experimental system. The manuscript is also generally well written and easy to understand.

# Referee report: Reviewer #2 (Public review)

**DOI:** 10.7554/eLife.110352.3.sa2

Summary:

In this paper, the authors set out to better understand the genetic mechanisms underlying thermal adaptation in insects. They experimentally evolved diamondback moth (Plutella xylostella) populations - a pest species with a wide distribution - under both hot (12h:12h 32{degree sign}C/27{degree sign}C) and cold (15{degree sign}C/10{degree sign}C) thermal conditions, and conducted phenotypic assays and metabolic and transcriptomic profiling to analyze how populations changed to deal with this thermal stress compared to the nonevolved ancestral population (constant 26{degree sign}C). Phenotypic assays showed that evolved hot populations had increased survival at high temperatures (42-43{degree sign}C) while evolved cold populations had lower freezing points compared to the ancestral population. When measured at the constant 26{degree sign}C conditions, metabolic and transcriptomic profiles of 3rd instar larvae from the evolved population were distinctive from the ancestral population, with a set of overlapping metabolic and transcriptomic pathways that were significantly differentially expressed in both hot and cold evolved populations compared to the ancestral. The authors narrowed down this set of candidate genes further by focusing on genes with high expression levels overall, whose expression profile was correlated with differentially expressed metabolites, and that contained mutants in both hot and cold strains. From this set, they chose the PxSODC gene for further functional validation, as it has previously been shown to be involved in the response of insects to abiotic stress with its antioxidative role in cellular defense. At the constant 26{degree sign}C, this gene showed lower expression across development in evolved strains compared to the ancestral population, while it showed similar expression patterns under thermal stress. Knockdown of PxSODC resulted in decreased survival rates at high temperatures and higher freezing points compared to the ancestral population. Based on this validation, the authors hypothesize that the non-synonymous mutation in the PxSODC gene that they found in the cold and hot evolved populations might alter the conformation of the PxSODC protein, increasing enzyme capacity. Their experimental evolution experiment furthermore indicates the capacity of the pest species, the diamondback moth, to adapt to a wide range of temperatures, providing insights into its capacity for global dispersal.

Strengths:

(1) The authors did a tremendous amount of work to characterize the mechanisms underlying thermal adaptation in the diamondback moth, artificially selecting populations for three years in the lab and characterizing how they evolved as a result at different biological levels: from phenotypes in different life stages, to larval metabolites and gene transcription, to functionally validating how one of the resulting gene candidates influences the capacity to deal with thermal stress.

(2) The paper identifies and provides further evidence for candidate genetic mechanisms that might be particularly important for thermal adaptation in insects, including lipid metabolism, oxidoreductase activity, and DNA methylation. It is furthermore interesting that the authors found similar mechanisms to be involved in both the adaptation to cold and hot environments. Their functional validation of some of the genes involved in these mechanisms is very useful to understand how these genes might be causally involved in insect thermal adaptation.

(3) The paper also has applied value: the diamondback moth is a pest species with a wide distribution, so understanding its adaptive capacity to different thermal environments is important for predicting the prevalence and potential further range expansion of this species under future climate change.

# Author comment: Author response

**DOI:** 10.7554/eLife.110352.3.sa3

**Authors:** Gaoke Lei, Huiling Zhou, Zongyao Ma, Yating Duan, Yanting Chen, Fengluan Yao, Minsheng You, Liette Vasseur, Geoff M Gurr, Shijun You

**Affiliations:** Fujian Academy of Agricultural Sciences Fuzhou China; Fujian Agriculture and Forestry University Fuzhou China; Fujian Agriculture and Forestry University Fuzhou China; Fujian Agriculture and Forestry University Fuzhou China; Fujian Academy of Agricultural Sciences Fuzhou China; Fujian Academy of Agricultural Sciences Fuzhou China; Fujian Agriculture and Forestry University Fuzhou China; Brock University Catharines Canada; Charles Sturt University Orange Australia; Fujian Agriculture and Forestry University Fuzhou China

The following is the authors’ response to the original reviews.

**eLife Assessment**

This important study deepens our understanding of how populations of a given species may diverge in their molecular and physiological patterns as a result of adaptation to different thermal regimes. By approaching this question from multiple directions, the authors provide solid evidence for adaptive changes in three strains of the diamondback moth after only three years of experimental evolution, and support the causal involvement of the PxSODC gene in thermal adaptation to both cold and hot temperatures. This work would benefit from more sophisticated phylogenetic analyses, better statistical support, and a more detailed discussion of the differences in the three strains at the pathway level.

We sincerely thank the editors for this positive and constructive assessment. In the revised manuscript, we have addressed the highlighted points by: (1) re-inferring the phylogenetic tree of the *PxSODC* gene using a model-based Maximum Likelihood method (IQ-TREE) to ensure a robust evolutionary analysis; (2) substantially expanding the description of our statistical methods across all data types to ensure reproducibility and clarify multiple-testing corrections; and (3) adding a more detailed discussion of the pathway-level differences between the hot and cold strains, particularly integrating how their distinct transcriptomic responses align with their shared metabolic adjustments and phenotypic traits.

**Reviewer #1 (Public review):**

(1) The authors identify pathways that are enriched in different strain comparisons (Figure 3E), but do not provide a detailed interpretation of these results. It would be great if the authors could explain in more detail how the physiological processes of a cold-adapted strain of this species may differ from those of a warmer-adapted strain.

We agree. We have addressed this by directly integrating our pathway enrichment results (Figure 3E) with the observed life-history phenotypes (concurrently addressing Reviewer 2's Comment 36a). We expanded the Discussion to explain that while both strains share convergent adjustments in core pathways (e.g., lipid metabolism for energy reallocation), their specific physiological strategies differ. The cold-adapted strain relies on broader transcriptional reprogramming to maintain homeostasis and support extended longevity/cold hardiness, whereas the hot-adapted strain utilizes broader metabolic rewiring to actively fuel its accelerated development and higher fecundity.

(2) The authors reconstruct a phylogenetic tree of the PxSODC gene using the neighbor-joining algorithm. The limitations of this algorithm have been known for many years now, especially for sequences separated by long evolutionary distances. According to Wang et al. (2016), the last common ancestor of the species shown in Figure S4C occurred 392-350 million years ago. Given this, I would strongly recommend that the authors infer a phylogenetic tree using model-based methods, such as those implemented in RAxML-NG or IQ-TREE. Also, in the absence of a valid outgroup sequence, I would show the gene tree as unrooted or rooted based on the corresponding species tree.

Agree. We have re-inferred the phylogenetic tree of the *PxSODC* gene using the model-based Maximum Likelihood (ML) method implemented in IQ-TREE. As recommended, in the absence of a valid outgroup sequence, the revised tree is now presented as unrooted. Supplemental Figure S4C (Figure 5-figure supplement 1C) and the corresponding text in the manuscript have been updated.

(3) There is a key piece of the puzzle that is currently missing: the structural mechanism behind the mutational effects described in this study (e.g., Figure 5). The authors could leverage AlphaFold to generate structural models of different mutants and conduct molecular dynamics simulations to examine their conformational dynamics.

We thank the reviewer for this excellent suggestion. We generated AlphaFold structural models of the wild-type (WT) and mutant (MU) PxSODC proteins and conducted 100 ns molecular dynamics (MD) simulations using GROMACS 2022.3 at three physiologically relevant temperatures: 15°C (cold stress), 26°C (favorable baseline), and 32°C (heat stress). Using 26°C as the physiological baseline, three key structural parameters support enhanced thermostability of the mutant protein (Figure 5–figure supplement 3). First, RMSD analysis revealed that under heat stress (32°C), the WT underwent severe conformational drift (RMSD increased from the 26°C baseline of 1.62 to 2.49, an increase of 0.87), while MU remained remarkably stable (from 1.59 to 1.66, an increase of only 0.07). Second, MU possessed a significantly more compact structure, with lower SASA values at 15°C (118.39 vs. 127.29 nm²) and 26°C (113.82 vs. 125.61 nm²), indicating optimized hydrophobic core packing. Third, the intramolecular hydrogen bond network of MU demonstrated dual stress resistance: under cold stress, MU actively increased hydrogen bonds from its baseline (113→119), whereas WT lost bonds (117→112); under heat stress, MU fully maintained its bond count (113→113). These results provide a direct structural mechanism for the enhanced catalytic efficiency of the mutant SOD at lower expression levels.

**Reviewer #1 (Recommendations for the authors):**

(4) The experimental evolution component of this study is described in the text as lasting for three years. It would help if the number of generations per strain were also reported.

We have added the number of generations per strain. Over the three-year period, the hot strain completed ~75 generations and the cold strain ~15 generations. The ancestral strain was continuously maintained at 26°C throughout this period. The revised text has been updated in both the Introduction and Materials and Methods.

(5) In Figure 3B: There is a typo in the word “Statistics”.

Corrected. The typo in “Statistics” in Figure 3B has been fixed.

(6) In Figure 3D: “CS” appears twice.

Corrected. The duplicated “CS” label in Figure 3D has been replaced with the correct label.

(7) Figure 4: This is not accessible to colorblind readers, who will clearly not be able to tell each color apart. As a non-colorblind person, I, too, have trouble figuring out which color label in panel B corresponds to which color in panel A. For example, I do not know off the top of my head how 'blue' differs from 'midnightblue', 'royalblue', or 'skyblue'. I recommend that the authors replace colors with identifiers, such as 'g1' for group 1 and so on.

We appreciate this suggestion. We have replaced all color-based module labels with alphanumeric identifiers (M1, M2, M3, etc.) and added a corresponding legend. The main text and supplementary materials have been updated accordingly.

(8) Lines 246-247: "Its secondary structure mainly consisted of strands, helices and coils." This sentence is redundant. These three are the only possible secondary structural elements, according to most bioinformatics tools such as PSIPRED, which the authors used. This sentence would be more useful if the authors could report the percentage breakdown of each secondary structural element.

We have removed the redundant sentence and updated the text to report the specific percentage breakdown of the secondary structural elements based on our PSIPRED predictions (approximately 55.24% random coils, 16.19% alpha helices, and 28.57% extended strands). The revised text has been updated in the Results section.

(9) Lines 260-261: "This suggests that the PxSODC gene can alter its expression pattern and function in response to environmental change...". I find this sentence a bit imprecise. Would it not be more precise to mention that the expression of this gene is regulated by temperature triggers?

We agree that the original phrasing was imprecise. We have revised the sentence in the manuscript to state: “This suggests that the expression of the *PxSODC* gene is regulated by temperature triggers, and its altered function contributes to temperature-adaptive evolution in *P. xylostella*.”

(10) The data points in Figures S1 and S7 are very small and hard to tell apart without zooming in a lot. Perhaps the authors could change the orientation of those pages to landscape and increase the size of the figures.

Done. We have changed the orientation of Supplemental Figures S1 (Figure 1-figure supplement 1) and S7 (Figure 5-figure supplement 4) to landscape and increased the size of the figures and individual data points to improve visibility.

(11) In Figure S2, the panel labeled as 'C' should be 'B' (based on the caption) and vice versa.

Corrected. The panel labels ‘B’ and ‘C’ in Supplemental Figure S2 (Figure 2-figure supplement 1) have been swapped. The Supplementary Materials have been updated accordingly.

**Reviewer #2 (Public review):**

(1) The paper in its current form is hard to digest and would benefit from improved clarification of the storyline, as well as a tighter integration between the phenotypic, omics, and functional validation data. Currently, it is not always clear what the relevance is of all the reported results, nor why certain decisions were made, or how all the different methods the authors used fit together. For example, the authors functionally validated a second gene, PxDnmt1, but it is unclear why this particular gene was chosen, nor how it relates to their selection regimes when looking at the results obtained with the phenotyping and omics data collection. Seeing how much work the authors did, this makes the paper overwhelming and difficult to read.

We sincerely appreciate this constructive feedback. In the revised manuscript, we have made significant structural revisions to improve the storyline and logical flow. We have streamlined the Results section (moving extensive descriptive data like life table curves and detailed metabolomics of mutant strains to the Appendix 1-3) to focus on the key findings. Furthermore, we have clarified the logical transitions between experiments. For instance, regarding the choice to validate *PxDnmt1*, we now explicitly explain in the Results that our untargeted metabolomic analysis of the *PxSODC* mutant strains revealed consistent alterations in 5-hydroxymethyluracil (involved in DNA demethylation) and 5'-deoxyadenosine (a precursor to the primary methyl donor S-adenosylmethionine) across all developmental stages. This specific metabolic signature provided a strong, data-driven hypothesis linking *PxSODC* function to epigenetic regulation via DNA methylation, prompting us to functionally validate *PxDnmt1*. By explicitly stating these rationales, the narrative is now much clearer and cohesive.

(2) The authors at times stretch their results too far, as the ecological relevance of their study design and results is not clear, limiting the generalizability and value of the results for understanding species' adaptive potential under climate change. For example, the selection regimes used present the minimum and maximum known temperatures at which the species can survive and develop, but it is unclear how the temperatures relate to the natural environment of the source population, to what extent wild populations might experience these temperatures, and whether they would experience them at the extended duration used (12h at max/min temperature). Moreover, I wonder whether the comparisons made would identify the genes that matter under natural conditions, as unevolved populations were kept under constant conditions compared to 12h:12h temperature regimes for the evolved populations, and the metabolic and transcriptomic profiling was done under a constant favorable 26°C rather than under thermal stress in a, as far as I can tell, randomly chosen life stage (larval stage).

We appreciate the reviewer raising these important points regarding ecological relevance and experimental design. In the revised manuscript, we have added context and acknowledged these limitations in the Methods and Discussion sections. First, regarding ecological relevance: The source population is from Fuzhou, a subtropical region where summer high temperatures frequently exceed 32°C and winter lows can drop below 10°C, making our selection temperatures ecologically relevant extremes for this population. The 12h:12h cycling temperatures were designed to simulate severe but natural diurnal fluctuations.

Second, regarding constant control vs. cycling regimes: The constant 26°C represents the established optimal developmental temperature and standard laboratory condition for *P. xylostella*. We acknowledge that comparing cycling selection regimes against a constant control might conflate adaptation to absolute temperature extremes with adaptation to thermal fluctuation itself. We have added this as a caveat in the Discussion. Third, regarding omics profiling conditions: The transcriptomic and metabolomic profiling was conducted under common garden conditions (26°C) specifically to identify constitutive, genetically fixed adaptations resulting from evolutionary selection, rather than immediate physiological plasticity under stress. We have clarified these rationales in the text.

(3) The paper in its current form does not adequately describe the statistical analyses underlying the results, nor do the authors share their code, making it very hard to judge whether the analyses used are appropriate and the results trustworthy. I have concerns about the inappropriate use of t-tests, the lack of correcting for confounding variables, and the need for multiple testing corrections.

We sincerely appreciate this concern. In the revised manuscript, we have made substantial improvements to the description of statistical analyses throughout the Methods section:

(1) Statistical methods for each data type are now described separately and in detail, specifying the tests used, the number and type of comparisons, and sample sizes.

(2) For metabolomic data, we have clarified that FDR correction was applied alongside multi-criteria thresholds (|log_2_Fold Change| ≥ 1, VIP ≥ 1, FDR < 0.05). For transcriptomic data, FDR correction (Benjamini and Hochberg, 1995) was applied via DESeq2.

(3) For WGCNA, we have specified the total number of correlation tests (29 modules × 30 metabolites = 870) and the stringent dual threshold (|*r*| > 0.8, *P* < 0.05) used to control for false positives, following standard practice.

(4) For life table parameters, the paired bootstrap method with 100,000 replications was used for all pairwise comparisons among strains.

(5) For all other experimental data (qRT-PCR, SOD activity, O_2_^-^ levels, survival rates, supercooling/freezing points, etc.), we have specified that t-tests were used only for two-group comparisons, while one-way ANOVA with Tukey's or Tamhane's T2 test was used for three or more groups, with non-parametric alternatives applied when normality assumptions were not met.

(6) The raw data have been deposited in public repositories (see Data availability), and all statistical procedures are now described in sufficient detail to enable independent reproduction of the results.

**Reviewer #2 (Recommendations for the authors):**

Title

(4) I don't feel the title adequately captures the work, I would instead of 'adaptive evolution' use 'experimental evolution' and I would not use the word 'underpins' but instead 'indicates', as it is not clear from your work whether the adaptations to the lab conditions you used would be ecologically relevant nor whether they are involved in thermal adaptation in wild populations.

Accepted. The title has been revised to: “Experimental evolution to thermal stress indicates climate resilience in a cosmopolitan arthropod.”

Abstract

(5a) Please add the phenotype results to the abstract.

We have added key phenotype results to the abstract. The revised text now reads: “The hot strain showed accelerated development, higher fecundity, and increased survival under extreme heat, while the cold strain exhibited lower supercooling and freezing points, indicating enhanced cold hardiness.”

(6b) The Abstract doesn't really detail the answer to your research question yet: so what insights into the genetic mechanisms underlying thermal adaptation did you gain that are novel?

We agree. We have revised the Abstract to explicitly highlight the novel genetic and molecular mechanisms we discovered. Specifically, we now detail that thermal adaptation is driven by a coordinated mutational, metabolic, and epigenetic (1) an energy-efficient genetic mechanism where non-synonymous mutations in *PxSODC* enhance superoxide scavenging efficiency, enabling effective oxidative stress management at lower gene expression levels; (2) convergent metabolic adjustments, notably a reduction in lipid metabolism to conserve energy; and (3) epigenetic regulation of thermal tolerance via DNA methylation. The revised text has been updated in the Abstract accordingly.

(7c) Line 3: replace 'ectotherms' with 'arthropods' to match the title?

Done. “Terrestrial ectotherms” has been replaced with “terrestrial arthropods” in the abstract.

(8d) Line 9: replace 'demographic' with 'life history'?

Done. “Demographic” has been replaced with “life history” in the abstract.

Introduction

(9a) The storyline is a bit unclear. Do you want to focus on the increased threat from insect pests under climate change or on the threat of climate change on insect persistence? Please pick one and adapt your storyline accordingly. I would suggest focusing on the first and talking more about the range extension of pest species under climate change (which would also require adaptation to cold extremes).

We agree and have refocused the Introduction on the increased threat from insect pests under climate change, emphasizing that range expansion into new regions requires adaptation to both heat and cold extremes. Both the first and second paragraphs have been revised accordingly.

(10b) Line 31-33: What do you mean by 'shows a positive relationship between the thermal tolerance range and the level of climatic variability'? Are they able to tolerate a larger range of temperatures?

This sentence has been revised as part of the restructured Introduction, which now focuses on the range expansion of pest species under climate change. The revised text reads: “Such range expansion requires adaptation not only to warmer conditions in existing habitats but also to cold extremes encountered during colonization of higher latitudes or elevations (Harvey et al., 2020).”

(11c) Line 33-35: Is this information relevant here?

Agreed. This sentence has been removed as part of the restructured Introduction, which now focuses on the threat of pest range expansion under climate change.

(12d) Line 55-56: What exactly do we not know yet about the mechanisms that enable thermal adaptation that you aim to fill in this paper? Please rephrase your knowledge gap to be more concrete (e.g., "but we do not yet know how...").

We have rephrased the knowledge gap to be more concrete and aligned with the revised storyline. The revised text now reads: “...we do not yet know how long-term thermal selection drives coordinated changes across gene function, metabolic networks, and life history traits to enable thermal adaptation and range expansion in pest species.”

(13e) Line 57: Also, here, the storyline is unclear. Why did you use the diamondback moth as your model species? You provide many different reasons, but it would help if you emphasized one reason that is in line with whichever storyline you want to focus on: is it because it is an insect pest that can tolerate a wide range of temperatures?

We have streamlined this paragraph to focus on the primary rationale: *P. xylostella* is a globally distributed pest that thrives across a wide range of thermal environments, making it an ideal model for studying the genetic mechanisms of thermal adaptation. Supporting details on genomic resources are retained briefly as they enable the multi-omics approach used in this study.

(14f) Line 65: Demonstrated how? Please give a short summary of the evidence for their genetic capacity to tolerate future climates.

We have added a brief summary of the evidence. Specifically, genome-wide SNP analysis of field populations from 114 locations across diverse biogeographical zones revealed climate-adaptive genetic variability, indicating that *P. xylostella* can tolerate projected future climates in most regions (Chen et al., 2021).

(15g) Line 72: What does 'Age-stage' mean? Should it read 'Aged-staged'?

“Age-stage, two-sex life table” is an established demographic method developed by Chi (1988) that simultaneously accounts for both age and developmental stage in both sexes. This is a standard term in the field (Chi et al., 2020), so we have retained the original wording but added a brief clarification upon first use.

(16h) Line 78-80: This needs a bit more explanation. Why does an increased ability to scavenge superoxide anions affect adaptability under extreme temperature environments?

We have added a brief explanation. Extreme temperatures induce oxidative stress by elevating intracellular reactive oxygen species (ROS), including superoxide anions, which can damage cellular structures. Enhanced scavenging capacity thus helps maintain cellular homeostasis under thermal stress.

(i) Line 82-86: Please be more precise. What novel insights did you gain about the genetic mechanisms underlying thermal adaptation?

We have revised this sentence to more precisely summarize the novel insights, encompassing both the multi-omics findings and the functional validation of *PxSODC*.

Results

(18a) The results section is very long and presents an overload of information at the moment, overwhelming the reader. Consider moving some sections to the Supplements (for example, a large part of the phenotypic data that cannot be linked to the omics data and the metabolic profiling of the mutant strains) or leave them out of the paper altogether.

We agree that the Results section was too dense. We have streamlined it by moving the following content to the Supplementary Materials:

(1) Detailed age-stage survival and fecundity curve data for the ancestral, hot and cold strains (Supplementary Text S1).

(2) Detailed life table analysis of the *PxSODC* mutant strains (Supplementary Text S2).

(3) Detailed untargeted metabolomic profiling of the SODC-MU mutant strains across developmental stages (Supplementary Text S3).

The main text now retains only the key life history comparisons, extreme temperature tolerance results, omics-based evidence linking transcriptomics and metabolomics, functional validation of *PxSODC*, and the DNA methylation findings, with brief summaries and cross-references to the Supplements for supporting details.

(19b) Please also provide the effect sizes for the different effects you report, for example, how many degrees difference was there between ancestral and cold strains in the supercooling/freezing points, and what was the variation?

We have added specific effect sizes (mean ± SEM and between-group differences) for all key comparisons throughout the Results section, including preadult duration, stage-specific survival rates under extreme heat, supercooling/freezing points, and SODC-MU mutant strain comparisons. For example, the supercooling points of CS pupae (-23.99 ± 0.18°C) were 0.90°C lower than AS (-23.09 ± 0.26°C), and the freezing points were 2.66°C lower (-14.24 ± 0.61°C vs. -11.58 ± 0.52°C). Please refer to the revised manuscript for all updated values.

(20c) Line 93-94: "Intrinsic and finite rate of increase" of what?

Clarified. These are population growth parameters. The revised text now specifies “intrinsic rate of increase (*r*) and finite rate of increase (*λ*) of the population.”

(21d) Line 98-99: Please start the paragraph with this summary of the results and then further detail them.

We have restructured this paragraph by moving the summary sentence to the beginning, followed by the supporting details.

(22e) Line 100-109: Why did you look at daily survival and fecundity rates? Please add why this is relevant.

As part of the overall streamlining of the Results section, this paragraph on detailed age-stage survival and fecundity curves has been moved to Supplementary Text S1. A brief justification for their relevance has been added there, noting that these curves capture stage-specific variation in survival and fecundity that summary life table parameters alone may obscure.

(23f) Line 106: What do HS, AS, and CS stand for? And please provide the statistics for comparison of daily survival rates between the strains.

We have defined the abbreviations (HS = hot strain, AS = ancestral strain, CS = cold strain) at their first appearance in the Results section. This paragraph on daily survival and fecundity has been moved to Supplementary Text S1, where the abbreviations are also defined. The survival rates reported are the maximum daily survival rates derived from the age-stage specific survival rate curves (*s_xj_*), and the statistical comparisons among strains are presented in Supplemental Table S1.

(24g) Line 144-146: Why are these differential metabolites likely to play a crucial role?

We agree this statement was speculative. It has been removed from the revised manuscript.

(25h) Line 159-161: Why is a reduction of lipid metabolites evidence for adaptive evolution?

We have revised this sentence to clarify the reasoning. The reduction in lipid metabolites in both independently evolved hot and cold strains suggests a convergent metabolic response, indicating that lipid metabolism adjustment is a shared adaptive strategy rather than a random change.

(26i) Line 184-185: It is difficult to judge from Figure 3E the extent of overlap in KEGG pathways between the hot and cold strains. Can you adjust the figure to emphasize that overlap more?

Agree. To intuitively emphasize the extent of overlap in KEGG pathways between the hot and cold strains, we have completely redesigned Figure 3E. Instead of presenting two separate panels with unaligned vertical axes, we have consolidated the data into a single back-to-back (mirrored) bar chart with a shared central y-axis.

(27j) Line 211: Not only the red module, but also the blue and green module correlates with many of the shared differential metabolites.

We agree. We have revised the text to acknowledge that the blue and green modules also showed strong correlations with shared differential metabolites, while noting that the red module had the highest number of significantly correlated metabolites and was therefore selected for further analysis.

(28k) Line 215: I would rephrase this as genes being interesting candidates for being involved in thermal adaptation or 'seem to be important for the adaptation of...', as you don't know from these results whether these genes play a critical regulatory role.

Agreed. We have toned down the language to reflect the correlative nature of these results.

(29l) Line 233: Do you mean that you further analyzed 15 genes of the 79 identified candidate genes in the previous paragraph?

Yes, exactly. From the 79 candidate genes, we selected 15 that were both annotated in the genome and had high expression levels (FPKM > 10) for further analysis. We have clarified this in the revised manuscript.

(30m) Line 238: What does SOD stand for?

We have spelled out the abbreviation upon first use in this section.

(31n) Line 254-255: Please provide the stats for this result.

We have added the specific allele frequencies for each strain. The Leu194-Met194 mutation frequency was determined by direct sequencing of 10 individuals per strain, and the frequencies are now reported in the revised text.

(32o) Line 303-304: How did you test for enhanced stability to temperature fluctuations? And enhanced compared to what?

This observation was based on the survival rate data in Figure 5C, where mutant pupae at 43°C showed no significant difference from the ancestral strain, whereas other life stages (eggs, larvae, adults) at 42°C showed significantly reduced survival in the mutant strains. We have revised the text to clarify the comparison.

(33p) Line 324-326: Why do decreased expression levels demonstrate increased O₂⁻ scavenging capacity? And why is that beneficial for adaptation to thermal stress? Please explain.

We have revised this sentence to clarify the logic. The non-synonymous mutations in the hot and cold strains likely alter the protein conformation of SOD enzymes, increasing their catalytic efficiency per molecule. This allows effective O_2_^-^ scavenging at lower expression levels, which is energetically favorable under thermal stress where energy conservation is critical for survival.

(34q) Line 404-406: I'm confused. Is there a direct link between the gene you knocked out here and the results you presented up until now? How do the reduced levels of 5-methylcytosine relate to the metabolite results you present at the beginning of the paragraph, other than that both could be involved in DNA methylation?

We have revised this paragraph to clarify the logical chain. Among the three metabolites consistently altered across all developmental stages in the SODC-MU strains, 5-hydroxymethyluracil is involved in dynamic DNA demethylation and 5'-deoxyadenosine is a precursor to S-adenosylmethionine (the methyl donor for DNA methylation). This suggested a link between *PxSODC* deletion and DNA methylation. To test this, we examined *PxDnmt1* expression and activity in the thermally adapted strains and found both were significantly reduced. We then used RNAi to silence *PxDnmt1* and confirmed that reduced DNA methylation (lower 5-mC levels) directly impaired thermal tolerance. Thus the connection is: *PxSODC* deletion → altered methylation-related metabolites → reduced DNA methyltransferase activity → decreased thermal tolerance.

(35r) Line 410: Saying that your knockdown of a gene that did not directly pop up in any of your other analyses confirms that DNA methyltransferase is associated with the response to thermal selection is a stretch. Please rephrase.

We agree this was overstated. We have toned down the language to reflect that the RNAi results provide preliminary evidence for a potential role of DNA methylation in thermal tolerance, rather than confirmation.

Discussion

(36a) The phenotype data are currently not discussed at all. Please add it to the discussion and try to integrate it more with the omics data you collected.

We agree. To provide a cohesive narrative and avoid redundancy, we have addressed this comment in conjunction with our pathway interpretation (please see our response to Reviewer 1, Comment 1). In the revised Discussion, we explicitly integrated our specific phenotypic findings (e.g., accelerated development, increased fecundity, and heat survival in the hot strain; prolonged lifespan and lowered supercooling points in the cold strain) with the distinct transcriptomic and metabolomic profiles. This integration demonstrates how molecular and metabolic rewiring directly underpins the divergent life-history traits without engaging in unwarranted speculation.

(37b) Line 433-434: I don't think this adequately represents the relevance of your particular study. I would suggest changing it to be more in line with the storyline of understanding the capacity for global dispersal in insect pests under climate change.

We agree. We have revised this sentence to align with the storyline of pest range expansion under climate change.

(38c) Line 476: This is a very odd statement; don't all species' genomes have genes encoding proteins involved in thermal adaptation? The reference also doesn't seem to be appropriate. I would suggest deleting this sentence.

Agreed. This sentence has been removed.

(39d) Line 483: Please write out SOD the first time you use it in a new section.

Done. SOD has been spelled out at its first use in the Discussion.

(40e) Line 544-548: This is a bit too specific to be the last sentence of the discussion. Try to formulate it more broadly in terms of what future research should focus on in general, not just your specific research.

We agree. We have broadened the final sentence to address future research directions more generally.

Figures

(41a) Figure 1A: I don't think t-tests are appropriate here since you are not simply comparing two treatments, but testing for the effects of 5-6 different temperatures. And how did you correct for replicate populations in your analysis?

Clarified. In Figure 1A, our comparisons are independent pairwise tests between exactly two strains (HS vs. AS) at each specific temperature and time point, making t-tests statistically appropriate. We were not testing for a continuous effect across temperatures. Regarding replicate populations, the individuals used in these assays were drawn from across the six replicate populations per treatment, with each biological replicate (n = 6, with 20 individuals per replicate) comprising individuals pooled from across the replicate populations to account for inter-population variation. We have clarified this in the revised figure legend.

(42b) Figure 1B, Figure 5D, Figure 7: bar graphs are used for count data, so do the data represent the number of individuals with a certain trait value? If they are instead showing the mean of the population/treatment group, please use mean points ± standard errors instead.

Accepted. The data in these figures represent continuous physiological traits (e.g., supercooling/freezing points) showing the mean of the populations, rather than count data. To align with current data visualization standards for continuous variables and to provide full transparency of the underlying data distribution, we have replaced the bar graphs in Figures 1B, 5D, and 7 with scatter plots. These revised figures now display the mean ± SEM overlaid with all individual biological replicate data points.

(43c) Figure 3B: There is a typo in the graph, it reads 'Stattistics' instead of 'Statistics'.

Corrected. The typo ‘Stattistics’ in Figure 3B has been fixed.

(44d) Figure 3C: I don't understand what the colors of the graph mean here. Is it the average differential expression of each replicate compared to the ancestral?

Clarified. We have updated the figure legend to explain that the colors represent the Pearson correlation coefficient (*r*) between pairs of biological replicates, indicating the degree of transcriptomic similarity among samples.

Methods

(45a) Please start each new methods paragraph with the purpose of the method/analysis, for example, "To investigate XX, we used method X to measure X". It is at the moment hard to understand why certain things were done.

We agree. We have revised each Methods paragraph to begin with a clear statement of purpose, so that the rationale for each analysis is immediately apparent. All changes are shown in the revised manuscript.

(46b) Line 575-578: Why were the selection regimes with cycling temperatures and the control with constant?

The cycling temperatures in the hot (32°C/27°C) and cold (15°C/10°C) regimes were designed to simulate diurnal temperature fluctuations (12h light/12h dark) that more closely reflect natural thermal environments. The control was maintained at a constant 26°C, which is the established optimal developmental temperature for *P. xylostella* (Liu et al., 2002) and represents the standard laboratory rearing condition. We acknowledge this asymmetry and have added a justification in the revised manuscript.

(47c) Line 581: How many generations was the ancestral population kept in the lab before the start of the selection experiment? And for how many generations were the populations selected?

The ancestral population was maintained in the laboratory for approximately ~170 generations (from July 2012 to the start of the selection experiment) before the thermal selection began. The hot strain was selected for ~75 generations and the cold strain for ~15 generations over the three-year experiment. We have added this information to the revised manuscript.

(48d) Line 585-586: I don't understand what you mean by randomly selecting six replicate populations per treatment for downstream experiments when you only had six replicate populations per treatment to begin with (as detailed in Line 574)?

We apologize for the confusion. All six replicate populations per treatment were used for downstream experiments. We have corrected this sentence to remove the misleading “randomly selected” wording.

(49e) Line 590: Were these 90 eggs also randomly selected, like for the individual life tables? And were these kept at the baseline temperature conditions?

Yes, the 90 eggs were randomly selected and maintained under the baseline favorable temperature (26°C). We have clarified this in the revised manuscript.

(50f) Line 606: Which life history and population fitness parameters were calculated?

We have specified all parameters calculated in the revised manuscript.

(51g) Line 609: Link to software doesn't work.

We have updated the software link to the current working URL.

(52h) Line 611: Please spell out what 'BT' stands for.

Done. “BT” has been spelled out as “bootstrap” upon first use.

(53i) Line 612-613: How many tests did you do? Did you correct for multiple testing? Using what method?

The paired bootstrap method implemented in TWOSEX-MSChart inherently accounts for multiple pairwise comparisons through 100,000 bootstrap replications. We have clarified the scope of comparisons in the revised manuscript.

(54j) Line 620-621: What does biological replicate mean here? Individual eggs / larvae / pupae / adults, or were all or some life stages pooled? Also, you now only detailed which samples were collected for metabolomic profiling, were the same samples used for transcriptomic profiling, or a subset?

Each biological replicate consisted of pooled individuals at the same developmental stage. The same sample collection strategy was used for both metabolomic and transcriptomic profiling, but from independent biological replicates (six for metabolomics, three for transcriptomics). We have clarified this in the revised manuscript.

(55k) Line 637: Also here, how many tests did you do? Were p-values corrected for multiple testing? Using what method?

Differential metabolites were identified through pairwise comparisons using Student's t-test with FDR correction for multiple testing. A multi-criteria threshold of |log_2_Fold Change| ≥ 1, VIP ≥ 1, and FDR < 0.05 was applied. This approach was used for all metabolomic comparisons, including HS vs. AS, CS vs. AS, and SODC-MU vs. AS. We have clarified this in the revised manuscript.

(56l) Line 662: And here: how many tests did you do? Did you correct for multiple testing? Using what method?

In the WGCNA analysis, Pearson correlations were calculated between each module eigengene and each of the 30 common differential metabolites, resulting in a total of 29 × 30 = 870 correlation tests. Following standard WGCNA practice, rather than applying FDR correction, we used a stringent dual threshold of |correlation coefficient| > 0.8 and *P* < 0.05 to identify significant module-metabolite associations, which effectively controls for false positives (Langfelder and Horvath, 2008). We have clarified this in the revised manuscript.

(57m) Line 663: How did you select these modules? The ones that significantly correlated with differential metabolites? Why did you not use the phenotype data here?

Modules were selected based on significant correlations (|correlation coefficient| > 0.8, *P* < 0.05) with differential metabolites shared between the hot and cold strains. We chose metabolites rather than phenotype data as the trait input for WGCNA because metabolites serve as intermediate molecular phenotypes that bridge gene expression and organismal phenotypes, providing a more direct link to the underlying regulatory mechanisms. This approach allowed us to identify gene modules most closely associated with the metabolic changes driven by thermal adaptation, which could then be connected to the observed life history and fitness divergence.

(58n) Line 666: move RNA extraction details to before RNAseq methods description.

Done. The “RNA extraction and cDNA synthesis” section has been relocated to before the “Transcriptomic profiling” section for better logical flow.

(59o) Line 836: This paragraph describing the statistics is very short, and it is unclear to what data the described analyses apply. As the different types of data are very different, I expect the analyses to differ as well. Please describe the statistical analyses for each data type in more detail, specifying what tests you used, which, and how many comparisons were performed.

We agree. The statistical methods for life table analysis, metabolomics, and transcriptomics have been detailed in their respective method sections. We have expanded the Data analysis section to specify the statistical tests for the remaining experimental data.

(60p) Line 837: Please include your SPSS scripts to ensure the reproducibility of your results.

The statistical analyses in SPSS were performed using the graphical user interface. As all statistical tests, parameters, and comparison groups have been described in detail in the revised Methods section, and the raw data have been deposited in public repositories (see Data availability), we believe the analyses are fully reproducible. We are happy to provide additional details if needed.
